# Relationship between alcohol use and overactive bladder disease: a cross-sectional study of the NHANES 2005–2016

**DOI:** 10.3389/fpubh.2024.1418117

**Published:** 2025-01-03

**Authors:** Yixin Zhang, Weijun Qin

**Affiliations:** Department of Urology, Xijing Hospital, Air Force Medical University, Xi'an, China

**Keywords:** OAB, NHANES, alcohol consumption, LUTS, urinary incontinence

## Abstract

**Background:**

Overactive bladder syndrome (OAB) is a prevalent urological condition which has a substantial impact on the life quality of affected individuals, resulting in restrictions in daily activities and work productivity. Alcohol is a diuretic, which means that it increases urine production and can potentially worsen urinary urgency and frequency. Several studies have investigated the association between alcohol consumption and OAB symptoms, but the results have been conflicting. This study aimed to investigate the relationship between alcohol consumption and OAB symptoms using a large, nationally representative sample.

**Method:**

Data from the National Health and Nutrition Examination Survey 2005–2016 were obtained for analysis. The Overactive Bladder Symptom Scale (OBSS) was used to determine the presence of OAB in each participant. Multivariate logistic regression and ordinal logistic regression were used to analyze the association of alcohol use frequency and quantity with the onset and severity of OAB, respectively.

**Results:**

A total of 7,805 samples (representing the 1,473,525,341 US population after weighting) were included in our analysis. Approximately 12.4% of this weighted sample self-reported having OAB. A greater proportion of nondrinkers, a higher proportion of females, higher blood pressure, older age, and lower income levels were observed in OAB patients compared to non-OAB patients. Univariate logistic regression revealed that the risk of OAB was significantly greater in the nondrinker group than in the 1–5 drinks/month (OR 0.64; 95% CI, 0.50–0.83), 5–10 drinks/month (OR 0.60; 95% CI, 0.43–0.82) and 10+ drinks/month groups (OR 0.41; 95% CI, 0.30–0.56) and the risk of OAB in the lowest quartile of alcohol consumption quantity was significantly higher than the second (OR 0.58; 95% CI, 0.47–0.70), third (OR 0.49; 95% CI, 0.39–0.62), and highest quartiles groups (OR 0.58; 95% CI, 0.45–0.75). The adjusted model revealed that only patients in the 10+ drinks/month group had a significantly lower risk of OAB than did those in the nondrinker group (OR = 0.64; 95% CI = 0.45–0.92), while the other two groups had similar risks. Furthermore, no significant association was found for the highest quartiles in the adjusted model; however, the second and third quartiles of alcohol consumption quantity group still exhibited obvious associations. These findings suggest that higher alcohol consumption, when appropriate, is associated with a lower risk of OAB compared to nondrinkers and the lowest quartile of alcohol consumption quantity group, even after adjusting for age, sex, race, and comorbidities.

**Conclusion:**

In conclusion, our findings revealed a significant association between alcohol consumption and the incidence of OAB in the study population. In terms of long-term effects, alcohol may not be a risk factor for OAB. These factors may represent intervention targets for lowering the risk and severity of OAB symptoms, but this needs to be confirmed in large clinical trials.

## Introduction

Overactive bladder (OAB) is a prevalent urological condition characterized by symptoms such as urgency, frequency, and nocturia, with or without urinary incontinence, in the absence of urinary tract infections or other identifiable causes (1). This condition significantly impacts the quality of patients’ life, often causing embarrassment, inconvenience, and disruption to daily activities. With increasing social stress increasing and lifestyle habits changing, the prevalence of OAB has risen in recent years. The exact etiology of OAB remains complex and multifactorial, involving both physiological and psychological factors. Emerging evidence suggests that not only biological factors such as benign prostatic hyperplasia (BPH) but also modifiable lifestyle factors may influence lower urinary tract symptoms (LUTS) (2, 3). Recent research has elucidated diverse facets of OAB. Studies have underscored the significance of bladder dysfunction, such as detrusor overactivity, as a pivotal mechanism in the pathophysiology of OAB (4, 5). Neurological factors, including alterations in sensory signaling and central nervous system dysfunction, have also been implicated in the development of OAB (6). Furthermore, hormonal imbalances, pelvic floor dysfunction, and genetic predisposition have been suggested as potential contributors to OAB (7).

Alcohol consumption has long been suspected to play a role in the development and exacerbation of OAB symptoms (8–10). Alcohol is a diuretic, which means that it increases urine production and can potentially worsen urinary urgency and frequency. Several studies have investigated the association between alcohol consumption and OAB symptoms, but the results have been conflicting. Some studies have reported a positive association between alcohol intake and OAB symptoms (8), while others have found no significant relationship (9).

Due to the limited availability of research on the relationship between alcohol consumption and OAB, most of the existing studies are cross-sectional observational studies (11, 12). It is important to note that some of these investigations were underpowered, meaning that they had a small sample size or insufficient statistical power to draw definitive results or conclusions. Therefore, further research, including larger and more rigorous studies, is needed to provide more conclusive evidence on the relationship between OAB and individual alcohol use status.

In this study, we aimed to investigate the relationship between alcohol consumption and OAB symptoms using a large, nationally representative sample. By analyzing data from the National Health and Nutrition Examination Survey (NHANES), we hope to provide valuable insights into the potential role of alcohol in the development and exacerbation of OAB symptoms.

## Method

### Data source and study population

The data for this study were obtained from 6 2-year cycles of the National Health and Nutrition Examination Survey (NHANES) conducted between 2005 and 2016. These specific years were chosen because they included questions about alcohol use and urination symptoms. NHANES is a comprehensive, ongoing survey conducted by the Centers for Disease Control and Prevention in the United States. It combines interviews and physical examinations to assess the health and nutritional status of individuals across different age groups. NHANES utilizes a complex, multistage, stratified, clustered probability sampling design to ensure that the collected data is representative of the entire U.S. population. For this study, we included participants aged >20 years who completed both the Kidney Conditions Questionnaire and the Alcohol Use questionnaire.

### Measurement of alcohol use

Alcohol use condition was obtained from the Alcohol Use questionnaire. The Alcohol consumption questionnaire was administered at the Mobile Examination Center (MEC), during the interview. This questionnaire is specifically designed for adults aged >20 years. Trained interviewers the questionnaire using the Computer-Assisted Personal Interviewing (CAPI) system, which ensures standardized and accurate data collection by guiding the interviewers through the questionnaire and recording the responses electronically. The assessment was based on the following questions answered by the participants:

1. Have you had at least 12 drinks of any type of alcoholic beverage? By a drink, I mean a 12 oz. beer, a 5 oz. glass of wine, or one and half ounces of liquor?
2. Have you had at least 12 drinks of any type of alcoholic beverage?
3. How often did you drink any type of alcoholic beverage?
4. In the past 12 months, on those days that you drank alcoholic beverages, on the average, how many drinks did you have?

The alcohol consumption status of the enrolled patients was obtained through the above four questions, and patients were grouped based on their alcohol consumption frequency. The monthly alcohol consumption was classified into four quartiles (Q1, Q2, Q3, and Q4) due to its skewed distribution.

### Measurement of overactive bladder symptoms

The assessment of OAB symptoms was obtained from the Kidney Conditions-Urology Questionnaire. This section presents personal interview data regarding kidney disease, kidney stones, urinary incontinence, and nocturia. Trained interviewers were asked about urinary incontinence and nocturia in the MEC using the CAPI system. The assessment of OAB symptoms was conducted by asking the participants the following questions:

1. Have you leaked or lost control of even a small amount of urine with an urge or pressure to urinate, and you could not get to the toilet fast enough?
2. How frequently does this occur?
3. How many times per night did you most typically get up to urinate, from the time you went to bed at night until the time you got up in the morning?

The frequency of urinary incontinence was calculated based on the first and second questions, while the frequency of nocturia was determined using the third question.

To assess OAB symptoms, we utilized a widely recognized questionnaire called the Overactive Bladder Symptom Score (OABSS), which was developed by Blaivas et al. (13), also as utilized by Shenhao Zhu et al. (14). The scoring criteria for this questionnaire are provided in Table 1. By summing the scores for nocturia and urge urinary incontinence, we obtained the OABSS for each participant in the NHANES study. A total score of ≥3 indicated a diagnosis of OAB (14).

**Table 1:** Criteria for OABSS score.

Urge urinary incontinence frequency	Urge urinary incontinence score
Never	0
Less than once a month	1
A few times a month	1
A few times a week	2
Every day or night	3
Nocturia frequency	Nocturia score
0	0
1	1
2	2
3	3
4	3
5 or more	3

NHANES, National Health and Nutrition Examination Survey; OABSS, Overactive Bladder Symptom Score.

### Other covariate measurements

Demographic variables. The NHANES demographic files included self-reported age, gender, race, family income-to-poverty ratio, education level, and marital status at the time of the interview.

Body mass index. The body mass index (BMI) data of the study participants were documented in the NHANES body measurements dataset. These measurements were conducted by proficient health technicians in the Mobile Examination Center (MEC).

Diabetes mellitus. Participants who met any of the following criteria were diagnosed with diabetes (15): (1) fasting glucose (mmol/L) ≥7.0, (2) had a random blood glucose level (mmol/L) ≥11.1, (3) had a glycohemoglobin HbA1c level (%) >6.5, (4) had a 2-h oral glucose tolerance test (OGTT) blood glucose level (mmol/L) ≥11.1, (5) had used antidiabetic agents, and (6) were told that they had diabetes by doctors.

The abovementioned information was obtained from the diabetes file, Oral Glucose Tolerance Test, Standard Biochemistry Profile, Plasma Fasting Glucose file, and Glycohemoglobin file.

Hypertension. In NHANES, hypertension was defined as self-reported hypertension by the participant (who responded “yes” to the question “Have you ever been told by a doctor or other health professional that you had hypertension, also called high blood pressure?”) or elevated blood pressure during the physical examination (mean systolic blood pressure ≥ 140 mm Hg or mean diastolic blood pressure ≥ 90 mm Hg) (16).

Metabolic syndrome. Metabolic syndrome was defined following the methodology outlined in previous literature (17, 18).

### Statistical analysis

The statistical packages R (version 4.1.2) were used for all the statistical procedures. Considering the intricate sampling design employed by NHANES, we incorporated strata, primary sampling units, and sampling weights in our data analysis, as per the guidelines provided by the National Center for Health Statistics, to derive estimates for the entire population of the United States. We integrated the samples of 6 2-year cycles from 2005 to 2016 for adult patients aged >20 years. In our study, OGTT (Oral Glucose Tolerance Test) data was employed for the purpose of screening diabetic patients, so we took one-sixth of the OGTT Subsample 2 Year MEC Weight (variable WTSOG2YR in demographics file) to calculate the combined weight of each individual. Statistical analyses were also conducted to determine the statistical disparities between individuals who regularly used alcohol and those who did not, considering both categorical and continuous variables. The chi-square test and t test were employed for categorical and continuous variables, respectively. To evaluate the correlation between alcohol use and the onset of OAB, univariate and multivariate logistic regression analyses were performed. Additionally, an ordinal logistic regression model was developed to examine the relationship between regular alcohol use and the severity of OAB. Model 2, Model3 and Model 4 employed multivariate logistic regression to account for the need to adjust for covariates, while Model 1 used univariate logistic regression. Model 1 was unadjusted; Model 2 adjusted for age and sex; Model 3 adjusted for age, sex, race, marital status, degree of education, and poverty income ratio; Model 4 adjusted for age, sex, race, marital status, economic standing, degree of education, BMI, waist circumference, hypertension, diabetes and metabolic syndrome.

## Results

**Figure 1:**
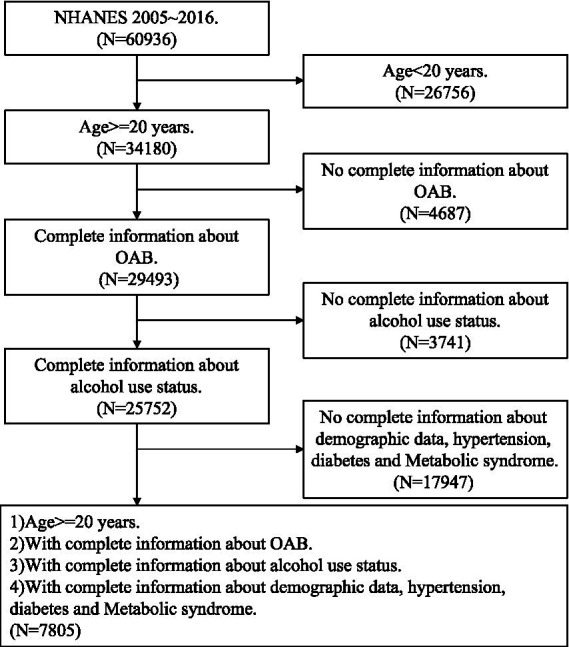
Flow chart.

After excluding participants who were under 20 years old (26,756) and lacked information on OAB symptoms (4687), alcohol use (3741), demographic data, hypertension data, and diabetes data (17947), a total of 7,805 participants (representing the 147,352,5,341 US population after weighting) were included in our analysis (Figure 1).

### Basic characteristics between OAB group and non-OAB group

The clinical characteristics of all included participants are shown in Table 2. The weighted sample size for overall patients was 1,473,525,341, of which 129,012,3,481 (87.6%) were in the non-OAB group and 18,340,1861 (12.4%) were in the OAB group. Characteristics such as Body Mass Index (BMI), sex, age, education level, race, marital status, FIPR (Family Income to Poverty Ratio) group, age group, glycohemoglobin level, HDL-Cholesterol level, triglyceride level, waist circumference, diabetes status, hypertension status, and metabolic syndrome status were analyzed. Individuals with OAB symptoms demonstrated a higher BMI (31 kg/m^2^) compared to those without symptoms (28 kg/m^2^), along with a notable gender distribution, with a greater proportion of females (59%) in the OAB group compared to the non-OAB group (46%). Moreover, the OAB group exhibited a higher mean age (55 years) compared to the non-OAB group (44 years). Additionally, disparities were observed in education level, race, marital status, FIPR group, age group, glycohemoglobin level, HDL-Cholesterol level, waist circumference, diabetes status, hypertension status, and metabolic syndrome status between the two groups, highlighting the multifactorial nature of OAB and its association with various demographic and clinical factors.

**Table 2:** Baseline characteristics of the study population according to OAB symptom status (NHANES 2005–2016).

Characteristic	Overall, *N* = 1,473,525,341	Weighted sample		*p*-value^2^
		Non-OAB group, *N* = 1,290,123,481	OAB group, *N* = 183,401,861	
BMI, kg/m^2^	29 (7)	28 (6)	31 (8)	<0.05
Sex				<0.05
Female	70,684,465 (48%)	59,869,022 (46%)	10,815,443 (59%)	
Male	76,668,069 (52%)	69,143,326 (54%)	7,524,743 (41%)	
Age, years	45 (16)	44 (15)	55 (16)	<0.05
Education level				<0.05
High school grad/GED or equivalent	30,781,978 (21%)	26,584,883 (21%)	4,197,095 (23%)	
Less than high school	18,858,783 (13%)	15,130,148 (12%)	3,728,635 (20%)	
More than high school	97,698,609 (66%)	87,287,539 (68%)	10,411,071 (57%)	
Race				<0.05
Mexican American	10,807,807 (7.3%)	9,460,761 (7.3%)	1,347,045 (7.3%)	
Non-Hispanic Black	14,682,256 (10.0%)	11,606,018 (9.0%)	3,076,239 (17%)	
Non-Hispanic White	106,192,440 (72%)	93,742,343 (73%)	12,450,096 (68%)	
Other Hispanic	6,887,359 (4.7%)	6,116,403 (4.7%)	770,956 (4.2%)	
Other Race – Including Multi-Racial	8,782,673 (6.0%)	8,086,823 (NA%)	695,850 (NA%)	
Marital status				<0.05
Living with a partner	13,519,492 (9.2%)	12,201,991 (9.5%)	1,317,501 (7.2%)	
Married	81,632,082 (55%)	71,771,703 (56%)	9,860,379 (54%)	
Single	52,200,960 (35%)	45,038,654 (35%)	7,162,306 (39%)	
FIPR group				<0.05
>5	42,480,547 (29%)	38,423,119 (30%)	4,057,428 (22%)	
≤1.0	18,098,907 (12%)	14,719,146 (11%)	3,379,761 (18%)	
1.1 ~ 5.0	86,773,080 (59%)	75,870,083 (59%)	10,902,997 (59%)	
Age group				<0.05
20–39 years	60,245,433 (41%)	56,739,859 (44%)	3,505,575 (19%)	
40–59 years	56,761,054 (39%)	49,818,242 (39%)	6,942,813 (38%)	
60–79 years	27,088,069 (18%)	20,541,450 (16%)	6,546,619 (36%)	
80+ years	3,257,978 (2.2%)	1,912,797 (1.5%)	1,345,180 (7.3%)	
Glycohemoglobin (%)	5.51 (0.83)	5.46 (0.75)	5.84 (1.23)	<0.05
HDL-Cholesterol, mg/dL	1.44 (1.16)	1.43 (1.14)	1.50 (1.30)	<0.05
Triglyceride, mg/dL	55 (16)	55 (16)	55 (17)	0.9
Waist Circumference, cm	98 (16)	97 (16)	104 (18)	<0.05
Diabetes				<0.05
Yes	17,164,776 (12%)	12,791,764 (NA%)	4,373,012 (NA%)	
No	130,187,758 (88%)	116,220,584 (90%)	13,967,174 (76%)	
Hypertension				<0.05
Yes	50,199,592 (34%)	39,944,312 (31%)	10,255,280 (56%)	
No	97,152,942 (66%)	89,068,037 (69%)	8,084,906 (44%)	
Metabolic syndrome				<0.05
Yes	49,449,873 (34%)	39,153,009 (30%)	10,296,864 (56%)	
No	97,902,661 (66%)	89,859,339 (70%)	8,043,322 (44%)	

^1^Mean (SD); n (%).

^2Wilcoxon rank-sum test for complex survey samples; chi-squared test with Rao & Scott’s second-order correction.^

### Alcohol use and overactive bladder symptoms

Table 3 presents the alcohol use status of the study population according to Overactive Bladder (OAB) symptom status. Significant differences were found between the non-OAB and OAB groups regarding alcohol consumption patterns. While non-drinkers constituted a higher proportion (12%) in the OAB group compared to the non-OAB group (7.4%), individuals in the OAB group demonstrated a slightly elevated frequency of consuming 1–5 drinks per month (56%) in contrast to the non-OAB group (55%). Moreover, although the mean alcohol quantity consumed was similar between the two groups (19 drinks), the distribution across alcohol quantity groups (Q1-Q4) revealed notable differences. Notably, a higher proportion of individuals in Q1 (37%) and Q3 (23%) was observed in the OAB group compared to the non-OAB group (24 and 31%, respectively). Then, according to the OABSS criteria, the frequency of patients consuming 5–10 drinks per month suffering from overactive bladder was lower than other patients (Table 4). Moreover, Table 5 also shows that according to alcohol consuming quantity, patients in group Q3 also exhibits a lower proportion of experiencing symptoms of overactive bladder compared to the other groups. The results indicate that with increasing alcohol consumption frequency, the proportion of individuals with overactive bladder (OAB) symptoms gradually decreases, reaching its lowest point in the group consuming alcohol 5–10 times per month, and then increases in the group consuming alcohol more than 10 times per month (Table 4). A similar trend is observed in alcohol consumption quantity, where the proportion of OAB patients decreases as alcohol consumption increases, reaching its minimum in group Q3, and then increases again in group Q4 (Table 5). These findings suggest potential associations between alcohol consumption patterns and OAB symptoms within the study population.

**Table 3:** Alcohol use status of the study population according to OAB symptom status (NHANES 2005–2016).

Characteristic		Weighted sample		*p*-value^2^
	Overall, *N* = 1,473,525,341	Non-OAB group, *N* = 1,290,123,481	OAB group, *N* = 183,401,861	
Alcohol frequency				<0.05
Non-drinker	11,671,179 (7.9%)	9,510,007 (7.4%)	2,161,172 (12%)	
1–5 drinks/month	81,086,959 (55%)	70,727,566 (55%)	10,359,393 (56%)	
5–10 drinks/month	19,985,664 (14%)	18,296,970 (14%)	1,688,694 (9.2%)	
10+ drinks/month	34,608,732 (23%)	30,477,805 (24%)	4,130,927 (23%)	
Alcohol quantity	19 (34)	19 (33)	19 (41)	<0.05
Alcohol quantity group				<0.05
Q1	38,210,643 (26%)	31,401,465 (24%)	6,809,178 (37%)	
Q2	33,221,175 (23%)	29,530,016 (23%)	3,691,159 (20%)	
Q3	44,272,979 (30%)	39,987,113 (31%)	4,285,866 (23%)	
Q4	31,647,737 (21%)	28,093,754 (22%)	3,553,983 (19%)	

^1^Mean (SD); n (%).

^2Wilcoxon rank-sum test for complex survey samples; chi-squared test with Rao & Scott’s second-order correction.^

Q1:<1 drink; Q2:1 ~ 6 drinks; Q3:6.25 ~ 24 drinks; Q4: >25 drinks.

**Table 4:** Overactive bladder symptom score according to alcohol use frequency (NHANES 2005–2018).

Characteristic		Weighted sample				*p*-value^2^
	Overall, *N* = 147,352,5,341	Non-drinker, *N* = 116,711,791	1–5 drinks/month, *N* = 810,869,591	5–10 drinks/month, *N* = 199,856,641	10+ drinks/month, *N* = 346,087,321	
Overactive bladder disorder						<0.05
Yes	18,340,186 (12%)	2,161,172 (19%)	10,359,393 (13%)	1,688,694 (8.4%)	4,130,927 (12%)	
No	129,012,348 (88%)	9,510,007 (81%)	70,727,566 (87%)	18,296,970 (92%)	30,477,805 (88%)	
Nocturia score						<0.05
0	56,047,184 (38%)	3,784,228 (32%)	32,139,137 (40%)	8,963,175 (45%)	11,160,644 (32%)	
1	59,444,088 (40%)	4,488,567 (38%)	31,366,185 (39%)	7,850,530 (39%)	15,738,806 (45%)	
2	21,168,073 (14%)	2,215,124 (19%)	11,379,728 (14%)	2,332,344 (12%)	5,240,878 (15%)	
3	10,693,189 (7.3%)	1,183,261 (10%)	6,201,908 (7.6%)	839,615 (4.2%)	2,468,405 (7.1%)	
Urge urinary incontinence score						<0.05
0	121,860,807 (83%)	8,512,325 (73%)	67,389,524 (83%)	16,828,533 (84%)	29,130,424 (84%)	
1	20,858,846 (14%)	2,400,419 (21%)	11,259,109 (14%)	2,643,306 (13%)	4,556,012 (13%)	
2	3,001,697 (2.0%)	459,585 (3.9%)	1,472,962 (1.8%)	390,144 (2.0%)	679,006 (2.0%)	
3	1,631,184 (1.1%)	298,851 (2.6%)	965,363 (1.2%)	123,680 (0.6%)	243,289 (0.7%)	
OABSS						<0.05
0	50,433,913 (34%)	3,155,234 (27%)	29,375,601 (36%)	7,888,342 (39%)	10,014,736 (29%)	
1	54,475,905 (37%)	3,849,871 (33%)	28,593,853 (35%)	7,598,041 (38%)	14,434,141 (42%)	
2	24,102,530 (16%)	2,504,903 (21%)	12,758,112 (16%)	2,810,587 (14%)	6,028,928 (17%)	
3	12,729,618 (8.6%)	1,297,078 (11%)	7,077,101 (8.7%)	1,302,640 (6.5%)	3,052,799 (8.8%)	
4	3,917,399 (2.7%)	517,612 (4.4%)	2,323,599 (2.9%)	240,607 (1.2%)	835,580 (2.4%)	
5	1,082,833 (0.7%)	215,511 (1.8%)	555,444 (0.7%)	133,546 (0.7%)	178,332 (0.5%)	
6	610,336 (0.4%)	130,970 (1.1%)	403,248 (0.5%)	11,901 (<0.1%)	64,216 (0.2%)	
OABSS level						<0.05
0	50,433,913 (34%)	3,155,234 (27%)	29,375,601 (36%)	7,888,342 (39%)	10,014,736 (29%)	
Mild (1 ~ 2)	78,578,435 (53%)	6,354,774 (54%)	41,351,964 (51%)	10,408,628 (52%)	20,463,069 (59%)	
Moderate (3 ~ 4)	16,647,017 (11%)	1,814,691 (16%)	9,400,700 (12%)	1,543,248 (7.7%)	3,888,379 (11%)	
Severe (5 ~ 6)	1,693,169 (1.1%)	346,481 (3.0%)	958,693 (1.2%)	145,446 (0.7%)	242,548 (0.7%)	

^1^n (%); Mean (SD).

^2Chi-squared test with Rao & Scott’s second-order correction; Wilcoxon rank-sum test for complex survey samples.^

**Table 5:** Overactive bladder symptom score according to alcohol use quantity (NHANES 2005–2018).

Characteristic		Weighted sample				*p*-value^2^
	Overall, *N* = 1,473,525,341	Q1, *N* = 382,106,431	Q2, *N* = 332,211,751	Q3, *N* = 442,729,791	Q4, *N* = 316,477,371	
Overactive bladder disorder						<0.05
Yes	18,340,186 (12%)	6,809,178 (18%)	3,691,159 (11%)	4,285,866 (9.7%)	3,553,983 (11%)	
No	129,012,348 (88%)	31,401,465 (82%)	29,530,016 (89%)	39,987,113 (90%)	28,093,754 (89%)	
Nocturia score						<0.05
0	56,047,184 (38%)	13,206,854 (35%)	13,581,112 (41%)	17,758,229 (40%)	11,500,989 (36%)	
1	59,444,088 (40%)	14,766,392 (39%)	12,958,958 (39%)	18,252,668 (41%)	13,466,070 (43%)	
2	21,168,073 (14%)	6,581,404 (17%)	4,315,541 (13%)	5,833,068 (13%)	4,438,060 (14%)	
3	10,693,189 (7.3%)	3,655,993 (9.6%)	2,365,563 (7.1%)	2,429,015 (5.5%)	2,242,618 (7.1%)	
Urge urinary incontinence score						<0.05
0	121,860,807 (83%)	28,611,095 (75%)	28,162,067 (85%)	38,046,856 (86%)	27,040,789 (85%)	
1	20,858,846 (14%)	7,587,307 (20%)	4,154,346 (13%)	5,188,674 (12%)	3,928,520 (12%)	
2	3,001,697 (2.0%)	1,169,559 (3.1%)	533,478 (1.6%)	793,408 (1.8%)	505,252 (1.6%)	
3	1,631,184 (1.1%)	842,682 (2.2%)	371,284 (1.1%)	244,041 (0.6%)	173,177 (0.5%)	
OABSS						<0.05
0	50,433,913 (34%)	11,417,594 (30%)	12,424,609 (37%)	16,140,212 (36%)	10,451,498 (33%)	
1	54,475,905 (37%)	12,696,671 (33%)	12,014,801 (36%)	17,121,542 (39%)	12,642,890 (40%)	
2	24,102,530 (16%)	7,287,200 (19%)	5,090,606 (15%)	6,725,359 (15%)	4,999,366 (16%)	
3	12,729,618 (8.6%)	4,328,117 (11%)	2,518,891 (7.6%)	3,274,198 (7.4%)	2,608,413 (8.2%)	
4	3,917,399 (2.7%)	1,643,863 (4.3%)	775,249 (2.3%)	770,832 (1.7%)	727,456 (2.3%)	
5	1,082,833 (0.7%)	502,413 (1.3%)	213,914 (0.6%)	209,736 (0.5%)	156,770 (0.5%)	
6	610,336 (0.4%)	334,786 (0.9%)	183,106 (0.6%)	31,100 (<0.1%)	61,344 (0.2%)	
OABSS level						<0.05
0	50,433,913 (34%)	11,417,594 (30%)	12,424,609 (37%)	16,140,212 (36%)	10,451,498 (33%)	
Mild (1 ~ 2)	78,578,435 (53%)	19,983,871 (52%)	17,105,407 (51%)	23,846,901 (54%)	17,642,256 (56%)	
Moderate (3 ~ 4)	16,647,017 (11%)	5,971,979 (16%)	3,294,140 (9.9%)	4,045,029 (9.1%)	3,335,869 (11%)	
Severe (5 ~ 6)	1,693,169 (1.1%)	837,199 (2.2%)	397,020 (1.2%)	240,836 (0.5%)	218,114 (0.7%)	

^1^n (%); Mean (SD).

^2Chi-squared test with Rao & Scott’s second-order correction; Wilcoxon rank-sum test for complex survey samples.^

Q1:<1 drink; Q2:1 ~ 6 drinks; Q3:6.25 ~ 24 drinks; Q4: >25 drinks.

### Ordered logistic regression model

**Figure 2:**
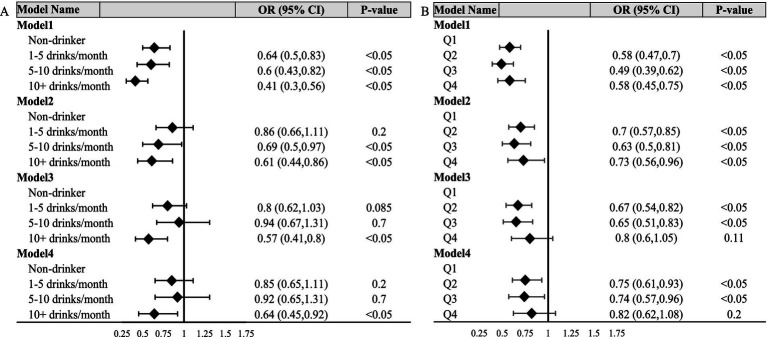
Forest polts. **(A,B)** Respectively depict the relationship between alcohol consumption frequency, alcohol consumption quantity, and OAB status. Model 1 showed the univariate analysis. Model 2 was further adjusted for age group and sex. Model 3 was further adjusted for BMI, poverty ratio, education level, race and marital status. Model 4 was further adjusted for diabetes and hypertension status. Q1: < 1 drink; Q2:1 ~ 6 drinks; Q3:6.25 ~ 24 drinks; Q4: >25 drinks.

To investigate the relationship between alcohol consumption frequency and the incidence of OAB in patients, our study conducted a univariate analysis, referred to as Model 1 (Figure 2A, Model 1), on the association between alcohol consumption frequency and disease incidence. Our crude model (adjusted for no variable) indicated a significant association between alcohol consumption frequency and OAB status. Compared with the non-drinker group, the risk of OAB was decreased in 1–5 drinks/month (OR = 0.64, 95% CI [0.5, 0.83], *p* < 0.05), 5–10 drinks/month (OR = 0.60, 95% CI [0.43, 0.82], *p* < 0.05) and over 10 drinks/month group (OR = 0.41, 95% CI [0.30, 0.56], *p* < 0.05), respectively. Simultaneously, after adjusted for age and sex (Figure 2A, Model 2), the two groups with higher frequencies still exhibited significant differences, while the 1–5 drinks/month showed no significant differences compared to the non-drinking group. Further adjusted for BMI, poverty ratio, education level, race and marital status (Figure 2A, Model 3), over 10 drinks/month group still exhibited significant differences compared to the non-drinking group (OR = 0.57, 95% CI [0.41, 0.80], *p* < 0.05), while no significant association was found for the other two groups. After additional adjusted for diabetes status and hypertension status (Figure 2A, Model 4), the group consuming over 10 drinks per month still demonstrated significant distinctions compared to the non-drinking group (OR = 0.64, 95% CI [0.45, 0.92], *p* < 0.05).

To explore the correlation between the quantity of alcohol consumption and the OAB status, we also constructed a crucial model and three adjusted models (Figure 2B). In summary, in both Model1 and Model2, it was observed that, relative to the lowest quartile of alcohol consumption quantity, the second, third, and highest quartiles groups exhibited a significantly reduced risk of OAB. In model3 and model4, no significant association was found for the highest quartiles, however, the second and the third quartiles of alcohol consumption quantity group still revealed obvious associations.

## Discussion

We utilized a nationally representative cross-sectional study to investigate the potential association between alcohol consumption and OAB in our research. The findings revealed a distinct correlation between alcohol intake and OAB status, suggesting that alcohol may not be a risk factor for this disorder. Moreover, exposure to alcohol consumption at high frequency and appropriate quantities might even reduce the risk of OAB.

OAB refers to a set of storage symptoms that significantly disrupt patients’ daily lives and emotional well-being, yet its underlying mechanisms remain unclear. Previous research (19–21) has suggested associations between OAB and neurological disorders, emotional changes, heightened sensitivity of bladder sensory nerve endings, increased spinal cord reflex excitability, and alterations in detrusor cell excitability or bladder mucosal epithelium neurotransmitter levels, all potentially contributing to the development of OAB.

Alcohol, scientifically known as ethanol, elicits diverse physiological responses upon ingestion. Following intake, ethanol is rapidly absorbed from the gastrointestinal tract into the bloodstream, where it permeates throughout the body (22). Through modulation of neurotransmitter activity, ethanol enhances gamma-aminobutyric acid (GABA) function while inhibiting glutamate, resulting in a dose-dependent depression of CNS function (23). These neurochemical alterations contribute to impaired cognition, diminished motor coordination, and attenuated inhibitions. Additionally, ethanol induces peripheral effects such as vasodilation, increased heart rate, and diuresis, further complicating its physiological impact (24). Chronic ethanol exposure may engender tolerance, dependence, and diverse adverse health outcomes, including hepatotoxicity, cardiovascular complications, and neurological impairments (25).

The impact of alcohol consumption has been extensively examined in several large-scale epidemiological studies. The EPINCONT study (26), as well as a comprehensive Italian study involving 5,488 subjects, both failed to establish any significant associations between alcohol or caffeine consumption and the observed outcomes (27). While existing data on the correlation between alcohol consumption and urinary incontinence are generally scarce, suggesting a tenuous link, our investigation unearthed a notable relationship between the frequency and quantity of alcohol intake and the onset of Overactive Bladder (OAB) within our study cohort.

The prevalence of OAB patients was notably higher among nondrinkers compared to other groups, but as alcohol consumption frequency increased, a discernible decrease in OAB incidence was observed. Therefore, in terms of long-term effects, alcohol may not be a risk factor for OAB. W. J. Bae et al. conducted a study revealing that moderate alcohol consumption has a mitigating effect on receptor and myogenic alterations in the bladder and that compared with nondiabetic rats, patients with moderate alcohol consumption exhibit greater tolerance to oxidative stress (28). In humans, alcohol is primarily metabolized into acetaldehyde by alcohol dehydrogenase (ADH) and further converted into acetate by acetaldehyde dehydrogenase (ALDH) and xanthine oxidoreductase (29). Most tissues in the body can metabolize alcohol (30). Acetaldehyde is a major contributor to the short- and long-term toxic effects associated with chronic alcohol consumption (31). Pathophysiological mechanisms involved in alcohol-related tissue and organ damage include oxidative stress, inflammation, and the formation of DNA adducts. It is believed that reduced fluid intake and dehydration can lead to bladder wall irritation due to the presence of highly concentrated urine, thereby increasing the risk of OAB and urge incontinence. The findings are consistent with observations from our study, indicating that patients with higher alcohol consumption exhibit a greater severity and incidence of overactive bladder (OAB) compared to those with minimal alcohol intake. Several studies have indicated that individuals experiencing urgency symptoms may intentionally consume less fluid as a means of self-managing their urgency and frequency symptoms (32). It is hypothesized that optimizing fluid intake, especially among the older adult demographic, may ameliorate symptoms associated with overactive bladder (OAB) and incontinence (33–35). An association between increased fluid intake, beyond the level necessary to quench thirst, and symptoms of OAB, such as urge urinary incontinence, has been reported, although limited evidence exists regarding this correlation (36–39). The present study and our study indicated that as the frequency and quantity of alcohol consumption appropriately increased among the population, the severity of OAB symptoms decreased, and fluid intake was shown to play a certain role in this relationship. According to previous research findings, the proportion of fluid intake attributed to alcohol may also provide support for these results.

Moreover, ingestible substances, particularly caffeine and opioids, have the potential to modulate the manifestations of OAB. Caffeine, an agonist of the central nervous system prevalent in beverages such as coffee, tea, and carbonated drinks, augments vigilance and stimulates diuresis (40). However, it may precipitate OAB-associated phenomena like increased urinary frequency and urgency, thereby posing considerable risks (41, 42). Conversely, opioids, renowned for their potent analgesic and sedative attributes, may exert deleterious effects on the urinary tract, including the suppression of bladder sphincter function and the induction of urinary retention. Regrettably, the present investigation encounters a significant limitation in the form of an inability to secure exhaustive data on both caffeine and opioid consumption from the NHANES repository, thereby emphasizing the necessity for additional prospective research endeavors to address and complement this area of inquiry.

This study has several strengths and limitations. The primary strength lies in its inclusion of a large population sample, which enhances the representativeness of the national population’s characteristics. Additionally, appropriate sampling weights were considered in the analysis to mitigate oversampling bias, thereby bolstering the reliability of our conclusions. This study also has certain limitations. First, this was a cross-sectional survey, precluding the establishment of a causal relationship between alcohol use and the occurrence of OAB. Second, the information provided by NHANES regarding OAB was incomplete, and the diagnosis of OAB relies primarily on questionnaire responses, thereby reducing the accuracy of the diagnosis. Finally, potential confounding factors that were not accounted for in the study cannot be determined.

## Conclusion

In conclusion, our findings revealed a significant association between alcohol consumption and the incidence of OAB in the study population. The proportion of OAB patients was significantly greater in the nondrinker group than in the drinker group, and as the alcohol consumption frequency increased, the proportion of OAB patients decreased. In terms of long-term effects, alcohol may not be a risk factor for OAB but rather a protective factor. The findings of this study may provide guidance for the daily lifestyle of OAB patients. Additional research is needed to more clearly define alcohol management for OAB.

## Data Availability

Publicly available datasets were analyzed in this study. This data can be found at: https://www.cdc.gov/nchs/index.htm.
